# Path-Routing Convolution and Scalable Lightweight Networks for Robust Underwater Acoustic Target Recognition

**DOI:** 10.3390/s25227007

**Published:** 2025-11-17

**Authors:** Yue Zhao, Menghan Chen, Yuchen Lu, Liangliang Cheng, Cheng Chen, Yifei Li, Nizar Faisal Alkayem

**Affiliations:** 1 School of Nautical Technology, Jiangsu Maritime Institute, Nanjing 211100, China; 2 Yantai Research Institute, Harbin Engineering University, Yantai 264005, China; 3 Engineering and Technology Institute Groningen, Faculty of Science and Engineering, University of Groningen, Nijenborgh 4, 9747 AG Groningen, The Netherlands; 4 College of Mechanics and Engineering Science, Hohai University, Nanjing 211100, China; 5 School of Engineering, Huzhou University, Huzhou 313000, China; 6 College of Automation, Nanjing University of Posts and Telecommunications, Nanjing 210046, China

**Keywords:** underwater acoustic target recognition, path-routing convolution, lightweight neural network, multi-scale feature extraction

## Abstract

**Highlights:**

**What are the main findings?**

- A novel PR-Conv mechanism is proposed to adaptively extract multi-scale ship acoustic features.
- The model achieves 98.58% classification accuracy while using 94% fewer parameters than conventional architectures.

**What is the implication of the main findings?**

- The lightweight design greatly reduces deployment cost and enables real-time inference.
- The model maintains a robust 77.8% accuracy under 10 dB SNR, demonstrating strong potential for real-world ship-radiated noise identification applications.

**Abstract:**

Maritime traffic surveillance and ocean environmental protection urgently require the accurate identification of surface vessel types. Although deep learning methods have significantly improved the underwater acoustic target recognition performance, the existing models suffer from large parameter counts and fail to adapt to the multi-scale spectral features of radiated noise from different vessel types, restricting their practical deployment on power-constrained underwater sensors. To address these challenges, this paper proposes a novel path-routing convolution mechanism that achieves the discriminative extraction of cross-scale acoustic features through multi-dilation-rate parallel paths and an adaptive routing strategy and designs the MobilePR-ConvNet unified architecture that enables a single framework to automatically adapt to diverse hardware platforms through systematic width scaling. Experiments on the DeepShip and ShipsEar datasets demonstrate that the proposed method achieved 98.58% and 97.82% recognition accuracies, respectively, while maintaining a 77.8% robust performance under 10 dB low-signal-to-noise-ratio conditions, validating the cross-dataset generalization capability in complex marine environments and providing an effective solution for intelligent deployment on resource-constrained underwater devices.

## 1. Introduction

The accurate identification of surface vessel types from underwater acoustic signals is critical for maritime safety and ocean monitoring [1,2]. As global maritime traffic intensifies and underwater autonomous systems expand, real-time vessel recognition has become essential for collision avoidance, illegal fishing detection, and marine protected area enforcement [3]. Traditional manual acoustic identification methods heavily rely on observer experience and subjective judgment and cannot process the massive data streams generated by modern hydrophone networks [4,5,6]. Therefore, automated recognition systems are urgently needed to enable intelligent maritime surveillance and autonomous underwater vehicle navigation [7,8].

Machine learning technologies have driven the paradigm shift in underwater acoustic target recognition from manual feature extraction to automatic feature learning [1,9]. Early methods primarily employed handcrafted features such as mel-frequency cepstral coefficients combined with backpropagation neural networks for classification [10,11,12], but these approaches suffered from cumbersome feature extraction processes and poor tolerance to marine environmental noise [13,14,15]. In recent years, deep learning methods have significantly improved the recognition performance by automatically extracting discriminative features from mel-spectrograms of ship-radiated noise using convolutional neural networks [16,17,18]. However, the existing deep models typically require millions of parameters and extensive computational resources, making them impractical for deployment on power-constrained underwater sensors and edge computing platforms [19,20,21].

The fundamental challenge lies in the multi-scale nature of ship acoustic signatures [22,23,24]. Large cargo vessels generate low-frequency broadband noise from diesel engines that spans wide spectral regions, while small tugboats produce localized high-frequency propeller harmonics [25]. Standard convolutions use fixed-size kernels that cannot adapt their receptive fields to these varying spatial scales. Consequently, networks must be made very deep or wide to capture all relevant features, leading to computational inefficiency that prevents real-world deployment on resource-constrained underwater platforms.

To address the limitation of fixed receptive fields, researchers have proposed various adaptive multi-scale feature extraction methods. Selective Kernel Networks (SKNets) [26] achieve dynamic receptive-field selection through parallel branches with different kernel sizes, but the significant parameter overhead from enlarging kernel sizes limits deployment on resource-constrained underwater devices; Atrous Spatial Pyramid Pooling (ASPP) [27] can expand receptive fields with fixed kernel sizes, but multi-path parallelism causes computational redundancy and lacks adaptive selection mechanisms; Pyramid Pooling Networks (PSPNets) [28] capture global context through spatial pooling, but downsampling operations lose fine-grained time–frequency structural information in ship-radiated-noise spectrograms. In summary, the existing multi-scale methods exhibit limitations in lightweight deployment, parameter efficiency, and adaptability to specific features of underwater acoustic signals.

The existing lightweight techniques address model complexity but fail to maintain recognition accuracy [29,30,31]. Network pruning and quantization reduce parameter counts yet degrade performance in low-signal-to-noise-ratio conditions common in ocean environments [32]. Mobile-optimized architectures like MobileNet lack mechanisms to selectively process the multi-scale spectral patterns inherent in vessel signatures. More critically, no unified framework exists to generate model families spanning IoT devices to desktop systems, forcing practitioners to redesign architectures for each deployment scenario.

To address these engineering challenges, we propose a novel path-routing convolution (PR-Conv) mechanism that enables adaptive receptive-field selection for ship acoustic recognition. PR-Conv constructs three parallel paths with dilated convolutions at rates of 1, 2, and 5 to capture local propeller transients, medium-scale engine harmonics, and global spectral envelopes, respectively, and then dynamically weights their contributions based on the input characteristics. The MobilePR-ConvNet family built upon PR-Conv directly targets the multi-scale feature extraction problem in underwater acoustics and supports network configurations from 0.25 times to 1.0 times through a unified-width-scaling mechanism, providing differentiated deployment solutions from IoT devices to desktop platforms without redesigning the recognition pipeline. The main contributions of this work are as follows:

1. We propose the PR-Conv mechanism, which addresses the problem of the significant cross-scale feature distribution differences in ship-radiated-noise spectrograms by achieving the discriminative extraction of acoustic features from different vessel types through multi-dilation-rate parallel paths and an adaptive routing strategy;
2. We design the MobilePR-ConvNet unified architecture, which addresses the deployment constraints of limited computational resources on underwater sensor nodes by achieving a lightweight recognition solution wherein a single framework automatically adapts to diverse hardware platforms through systematic width scaling;
3. We achieved 98.58% and 97.82% recognition accuracies on the DeepShip and ShipsEar datasets, respectively, while maintaining a 77.8% robust performance under 10 dB low-signal-to-noise-ratio conditions, validating the cross-dataset generalization capability and engineering applicability of the method in complex marine acoustic environments.

## 2. Proposed Method

### 2.1. Problem Definition

The underwater acoustic target recognition task can be formalized as a multi-class classification problem. Raw underwater acoustic signals are collected through sonar systems and record the radiated noise generated by different vessel types during underwater navigation. Let the original acoustic signal be a temporal sequence, $s(t)\in {\mathbb{R}}^{T}$, where T denotes the signal length. The objective of this study is to construct a mapping function ($f:{\mathbb{R}}^{T}\to C$) that maps acoustic signals to a discrete class space (C). This mapping process is realized through a deep neural network whose parameters (Θ) are optimized by minimizing the empirical risk during the training phase.

The recognition pipeline comprises two stages of feature transformation and classification decision. The collected raw signals are computed by the deployed device’s CPU through time–frequency transformation, $\phi :{\mathbb{R}}^{T}\to {\mathbb{R}}^{H\times W}$, into mel-spectrogram representations, where H and W denote the frequency and time dimensions, respectively. Subsequently, the deployed lightweight deep network ($g_{\Theta }:{\mathbb{R}}^{H\times W}\to {\mathbb{R}}^{\left|C\right|}$) extracts discriminative features and outputs a class probability vector, $p={\left[p_{1},p_{2},\ldots ,p_{\left|C\right|}\right]}^{⊤}$, where $p_{i}$ represents the probability that the signal belongs to the i-th class and satisfies $\sum_{i=1}^{\left|C\right|}p_{i}=1$. The final predicted class is determined by the maximum a posteriori criterion as $\hat{c}=\arg\max_{i}p_{i}$, achieving automatic surface vessel type recognition.

### 2.2. Mel-Spectrogram Feature Extraction

The discriminative features of vessel-radiated noise are primarily distributed in the frequency domain where different vessel types exhibit differentiated spectral energy distribution patterns in low-frequency bands. Therefore, this study employs mel-spectrogram transformation to realize time–frequency domain feature representation and maps the temporal signal (s(t)) into two-dimensional images suitable for convolutional network processing.

The short-time Fourier transform decomposes the temporal signal into time–frequency representation:

(1) $$X(m,k)=\sum_{n=0}^{N-1}x(n)w(n-m)e^{-j2\pi kn/N}$$

where x(n) is the discrete signal, w(n) is the window function, N is the window length, and m and k are the timeframe index and frequency index, respectively.

The mel filter bank converts the linear frequency to the mel scale through triangular filters:

(2) $$H_{i}(k)=\left\{\begin{array}{ll} \frac{k-f(i-1)}{f(i)-f(i-1)} & f(i-1)\leq k\leq f(i)\\ \frac{f(i+1)-k}{f(i+1)-f(i)} & f(i)\leq k\leq f(i+1)\\ 0 & \mathrm{otherwise} \end{array}\right.$$

where $H_{i}(k)$ is the frequency response of the i-th filter, and f(i) is the center frequency of the i-th filter.

The mel-spectrogram is obtained through the weighted summation of the power spectrum using the filter bank:

(3) $$S(m,i)=\sum_{k=0}^{N/2}|X(m,k){|}^{2}H_{i}(k)$$

where S(m,i) represents the energy at the m-th timeframe in the i-th mel-frequency band.

Logarithmic transformation compresses the dynamic range and enhances the visibility of weak signals:

(4) I=log(S+ϵ1)

where $I\in {\mathbb{R}}^{H\times W}$ is the final mel-spectrogram input, ϵ is a numerical stability constant, and 1 is an all-ones matrix.

### 2.3. Path-Routing Convolution

**Figure 1:**
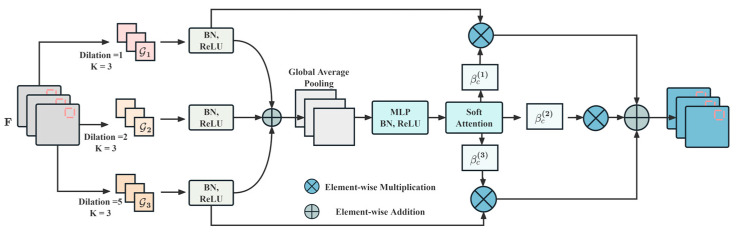
Schematic diagram of the principle of PRConv.

Different vessels exhibit significant scale variations in their radiated noise on spectrograms, where large vessels typically produce low-frequency broadband energy distributions, while small vessels that exhibit high-frequency narrowband characteristics and traditional convolutions with fixed receptive fields struggle to effectively capture these cross-scale discriminative patterns simultaneously. Addressing this multi-scale feature extraction problem, this study proposes the path-routing convolution mechanism, which constructs three parallel variable-receptive-field paths and adaptively allocates path weights based on input spectral features to achieve the adaptive recognition of acoustic signal features from different vessels. The design details of PRConv are shown in Figure 1.

#### 2.3.1. Multi-Path Feature Splitting

Vessel-radiated-noise spectrograms simultaneously contain local transient impact features and global persistent frequency band features, where a single-receptive-field scale cannot balance details and overall patterns [23]. Therefore, this study designs three parallel paths to extract local fine-grained textures, medium-scale spectral structures, and global energy distributions, constructing a multi-scale receptive-field spectrum from local to global through dilated convolutions, enabling mel-spectrogram features to enter convolution kernels of different scales for feature extraction.

For the input feature map ($F\in {\mathbb{R}}^{H\times W\times C_{in}}$), three parallel paths perform convolution transformations at different scales:

(5) $$F^{\left(1\right)}=T_{1}(F),\;F^{\left(2\right)}=T_{2}(F),\;F^{\left(3\right)}=T_{3}(F)$$

where $F^{\left(i\right)}\in {\mathbb{R}}^{H\times W\times C_{out}}$ is the output feature map of the i-th path.

The first path employs standard 3×3 convolution ($T_{1}$) to capture local fine-grained time–frequency textures in spectrograms for recognizing the transient pulse signal features generated by vessel propellers.

The second path employs dilated convolution ($T_{2}$) with the dilation rate ρ=2 to extract medium-scale spectral structural patterns for capturing periodic harmonic components generated by vessel engines.

The third path employs dilated convolution ($T_{3}$) with the dilation rate ρ=5 to capture large-scale global spectral distribution features for recognizing the broadband energy envelope of overall vessel-radiated noise.

#### 2.3.2. Global Feature Fusion

The multi-scale features extracted by the three paths need to be fused to obtain global statistical information on the input spectrum, providing a discriminative basis for the subsequent adaptive path selection. Element-wise addition fuses the feature responses from the three paths:

(6) $$G=F^{\left(1\right)}⊕F^{\left(2\right)}⊕F^{\left(3\right)}$$

where ⊕ denotes the element-wise addition, and $G\in {\mathbb{R}}^{H\times W\times C_{out}}$ is the fused feature map.

Global average pooling computes the average value of the spatial feature map for each channel, compressing the two-dimensional feature map into a one-dimensional vector to obtain the global response intensity statistics for each channel:

(7) $$\mu ={\left[\mu_{1},\mu_{2},\ldots ,\mu_{C_{out}}\right]}^{⊤},\;\mu_{c}=\frac{1}{H\times W}\sum_{h=1}^{H}\sum_{w=1}^{W}G_{c}(h,w)$$

where $\mu \in {\mathbb{R}}^{C_{out}}$ is the channel-level global statistical vector, and $\mu_{c}$ is the average activation value of the c-th channel across spatial dimensions.

Fully connected layers perform nonlinear transformation and dimensionality reduction on the channel statistical vector to generate compact feature representations:

(8) $$\xi =\sigma (\mathrm{BN}(W_{fc}\mu +b_{fc}))$$

where $\xi \in {\mathbb{R}}^{D}$ is the compact feature vector, σ is the ReLU activation function, BN is batch normalization, $W_{fc}\in {\mathbb{R}}^{D\times C_{out}}$ and $b_{fc}\in {\mathbb{R}}^{D}$ are learnable parameters, and $D=\max(\left\lfloor C_{out}/r\right\rfloor ,D_{min})$ is determined by the compression ratio (r) and minimum dimension ($D_{min}$).

#### 2.3.3. Adaptive Path Routing

Different vessels require receptive fields of different scales for feature extraction, where the broadband low-frequency features of large vessels rely on global paths, while the narrowband high-frequency features of small vessels rely on local paths. Therefore, this study designs an adaptive routing mechanism that dynamically generates attention weights for each path based on global feature vectors of the input spectrum, enabling the network to automatically select appropriate receptive-field combinations according to the spectral complexity of the current input vessel acoustic signals to achieve the discriminative feature recognition of acoustic targets.

Channel-level soft attention weights are computed through Softmax normalization:

(9) $$\alpha_{c}^{\left(i\right)}=\frac{\exp(\theta_{c}^{(i)⊤}\xi )}{\sum_{j=1}^{3}\exp(\theta_{c}^{(j)⊤}\xi )},\;i\in \{1,2,3\}$$

where $\alpha_{c}^{\left(i\right)}$ is the attention weight of the c-th channel on the i-th path, and $\theta_{c}^{\left(i\right)}\in {\mathbb{R}}^{D}$ is the learnable parameter vector, satisfying the normalization constraint $\sum_{i=1}^{3}\alpha_{c}^{\left(i\right)}=1$.

The output feature map performs the weighted aggregation of feature responses from the three paths through attention weights, completing the full recognition pipeline from mel-spectrogram input through multi-scale convolutional feature extraction to adaptive feature fusion:

(10) $$Y_{c}=\sum_{i=1}^{3}\alpha_{c}^{\left(i\right)}⊙F_{c}^{\left(i\right)}$$

where $Y_{c}\in {\mathbb{R}}^{H\times W}$ is the output feature map of the c-th channel, and ⊙ denotes element-wise multiplication.

### 2.4. Lightweight PR-ConvNet Architecture

**Figure 2:**
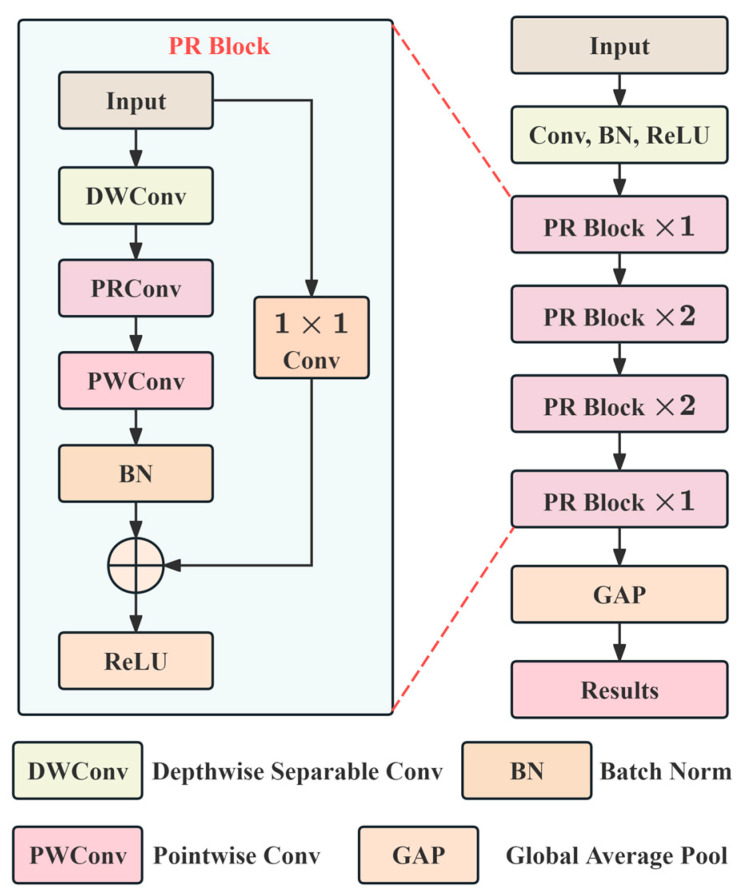
Overall architecture of MobilePR-ConvNet with four-stage hierarchical structure.

This study constructs four lightweight network architectures (MobilePR-ConvNet) with different complexities based on PR-Conv, implementing the adaptive configuration of the network parameters through a unified-width-scaling mechanism, enabling a single architectural design to automatically select appropriate channel configurations according to the computational resource constraints of target hardware platforms. The overall architecture of MobilePR-ConvNet is illustrated in Figure 2, where the network adopts a four-stage hierarchical structure, and each stage contains bottleneck blocks composed of depthwise separable convolution, the PR-Conv module, and pointwise convolution with stride convolutions between stages for feature map downsampling and residual connections spanning all blocks to ensure effective gradient propagation. The four architectural variants employ width multipliers (α∈{0.25,0.5,0.75,1.0}) to uniformly scale the baseline channel configuration [32, 64, 128, 256, 512], achieving full coverage deployment capability from IoT devices to desktop platforms while maintaining the core PR-Conv mechanism unchanged by adjusting the network width. This scalable design framework avoids the redundant work of designing separate network architectures for different hardware platforms, where users only need to select the corresponding width multiplier according to available computational resources to obtain the optimal balance between performance and efficiency.

The constructed MobilePR-ConvNet implements end-to-end recognition from mel-spectrogram input to vessel class output through the aforementioned four-stage structure, where the final stage employs global average pooling to compress feature maps and maps to the class space through fully connected layers outputting the probability distribution ($P(c_{i}|x)$) with the predicted class ($\hat{c}=\arg\max_{i}P(c_{i}|x)$) determined after Softmax normalization. The training process optimizes the network parameters using cross-entropy loss ($L=-\sum_{i}y_{i}^{\mathrm{true}}\cdot \log P(c_{i}|x)$), enabling PR-Conv to learn the discriminative multi-scale spectral patterns of different vessels.

## 3. Datasets and Evaluations

### 3.1. Dataset Description

This study employed the publicly available DeepShip underwater acoustic dataset for the comprehensive evaluation experiments [2]. The dataset collects radiated-noise signals from different types of vessels through an Ocean Sonics icListen AF hydrophone deployed at the Strait of Georgia Delta Node in British Columbia, Canada, where the sensor operates with a bandwidth from 1 Hz to 12 kHz, a dynamic range of 120 dB, and a sensitivity of −170 dBV re. 1 μPa, and the data collection spans from 2 May 2016 to 4 October 2018. The dataset contains 609 independent acoustic recordings with a total duration of 47 h and 4 min and a sampling frequency of 32 kHz covering four major commercial vessel types: Cargo, Passenger, Tanker, and Tug, where the different vessel types generate underwater radiated-noise signals with different spectral characteristics due to differences in their mechanical structures and propulsion systems. Specifically, cargo vessels generate stable low-frequency line spectra from large diesel engines, passenger vessels exhibit prominent high-frequency noise due to high-speed navigation, tankers display mixed characteristics of low-frequency line spectra and mid-frequency harmonics, while tugs produce broadband noise with strong modulation characteristics due to their frequent speed changes and high power output. These differentiated acoustic signatures of the four vessel types provide an ideal benchmark for validating the multi-scale feature extraction capability of PR-Conv. The data collection environment includes complex marine background noise tidal currents, biological activity, and human activity noise, providing a realistic and challenging testing platform for network robustness evaluation.

### 3.2. Hardware and Software Environment

Experiments were conducted on a high-performance computing platform with a hardware configuration including an Intel Core i9-14900HX processor NVIDIA GeForce RTX 4070 Laptop GPU and 32 GB DDR5-4800 memory, providing sufficient computational resources for deep learning model training and inference. The software environment was built on the PyTorch 2.0.1 deep learning framework running in the Python 3.9.18 and CUDA 11.8 environment, with the key dependency libraries including librosa 0.10.1 for audio signal processing, numpy 1.24.3 for numerical computation, and scikit-learn 1.3.0 for evaluation metric calculation. All experiments were executed on an Ubuntu 20.04 LTS operating system, ensuring experimental environment stability and reproducibility.

### 3.3. Data-Splitting Strategy

Each original acoustic recording was divided into fixed-length 3 s timeframe segments, obtaining 56,468 independent acoustic samples from the original 609 recordings. To avoid data leakage, this study strictly followed the record-level splitting principle, where all the timeframe segments from the same original recording are assigned to only one of the training or test sets, ensuring that the test set truly reflects the model generalization capability on unseen data. The training and test sets were split in an 8:2 ratio containing 45,174 and 11,294 samples, respectively.

### 3.4. Evaluation Metrics

This paper adopts accuracy as the primary evaluation metric:

(11) $$\mathrm{Accuracy}=\frac{\mathrm{TP}+\mathrm{TN}}{\mathrm{TP}+\mathrm{TN}+\mathrm{FP}+\mathrm{FN}}$$

where TP is the number of true positives, TN is the number of true negatives, FP is the number of false positives, and FN is the number of false negatives.

The macro-averaged F1-score is simultaneously adopted as a supplementary metric:

(12) $$F1\mathrm{-score}=2\times \frac{\mathrm{Precision}\times \mathrm{Recall}}{\mathrm{Precision}+\mathrm{Recall}}$$

where the precision ($\mathrm{Precision}=\frac{\mathrm{TP}}{\mathrm{TP}+\mathrm{FP}}$), recall ($\mathrm{Recall}=\frac{\mathrm{TP}}{\mathrm{TP}+\mathrm{FN}}$), and macro-averaged F1-score perform equal-weighted averaging across all class results.

Each experimental configuration independently executes 10 training–testing cycles using different random seeds for parameter initialization and data shuffling, with final reports presenting the arithmetic mean and standard deviation of all run results.

To evaluate the deployment feasibility of the proposed models on IoT and embedded devices, this paper measures model complexity metrics, including the number of parameters (Params) and floating-point operations (FLOPs), as well as practical deployment performance metrics, including the GPU inference time, model storage size (Size), CPU latency (Latency), single-core frame rate (FPS), CPU memory usage (CPU Mem), power consumption (Power), and CPU utilization (CPU Util). The Params and FLOPs reflect the theoretical computational complexity of the model, while the Size, Latency, FPS, CPU Mem, Power, and CPU Util directly measure the actual operational efficiency of the model on resource-constrained devices. Among these memory usage and power consumption metrics are critical metrics for evaluating the edge device deployment feasibility, directly determining the sustainable operational capability of models in battery-powered and memory-constrained scenarios. The comprehensive evaluation of these metrics can fully demonstrate the adaptability and deployment potential of the MobilePR-ConvNet-series models on different hardware platforms.

## 4. Results and Discussion

### 4.1. Classification Performance and Deployment Feasibility

This section comprehensively evaluates the classification performances of the proposed MobilePR-ConvNet-series models in vessel type recognition and their deployment feasibility on resource-constrained devices through training convergence dynamics confusion matrices and resource consumption metrics.

**Figure 3:**
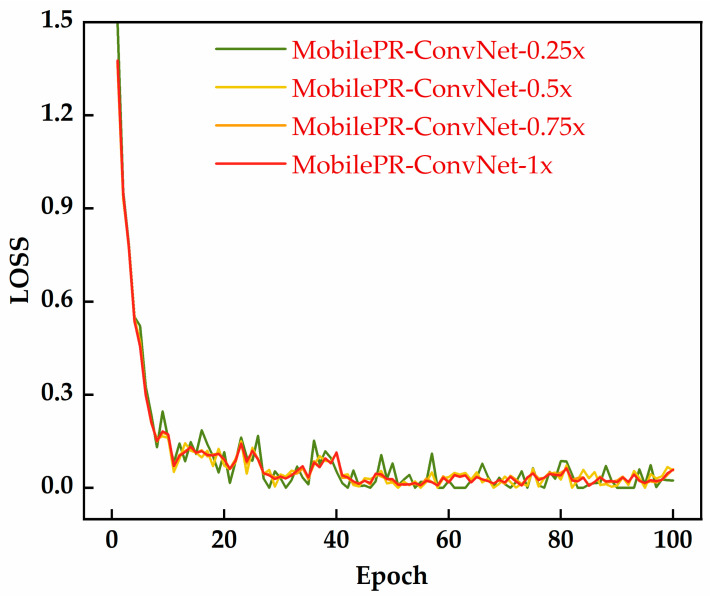
Training loss curves of four MobilePR-ConvNet variants during 100-epoch training process.

**Figure 4:**
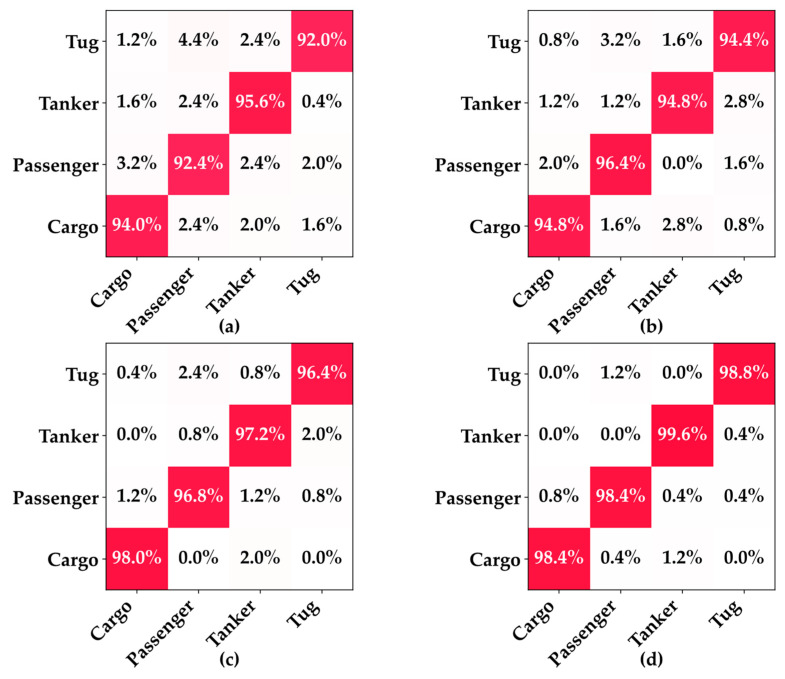
Confusion matrices of four MobilePR-ConvNet variants: (**a**) MobilePR-ConvNet-0.25×; (**b**) MobilePR-ConvNet-0.5×; (**c**) MobilePR-ConvNet-0.75×; (**d**) MobilePR-ConvNet-1.0×.

As shown in Figure 3, all four MobilePR-ConvNet variants exhibited stable convergence behavior during 100 training epochs, with loss values rapidly decreasing from 1.4–1.5 to 0.1–0.2 within the first 10 epochs. Among them, MobilePR-ConvNet-1.0× showed the smoothest convergence curve and minimal oscillations, benefiting from its larger network capacity and more comprehensive channel configuration. The confusion matrices shown in Figure 4 detail the classification performances of the four models in recognizing the surface vessel types through underwater acoustic signals, where all variants performed excellently on the four vessel types, Cargo, Passenger, Tanker, and Tug, with high diagonal values indicating a strong class discrimination capability. MobilePR-ConvNet-1.0× achieved the best overall performance, with the following class accuracies: Cargo: 98.4%; Passenger: 98.4%; Tanker: 99.6%; and Tug: 98.8%, validating the effectiveness of the standard width multiplier at maximizing the PR-Conv feature extraction capability.

A comprehensive analysis of the confusion matrices reveals that all models exhibit minimal inter-class confusion, with misclassifications mainly occurring between vessel types with similar acoustic characteristics. Analysis of the off-diagonal elements in the confusion matrices indicates that the few erroneous cases are primarily concentrated in mutual confusion between Cargo and Passenger, attributable to both being large commercial vessels with similar low-frequency mechanical noise characteristics, while Tanker demonstrates the highest recognition accuracy across all variants due to its distinctive cargo-hold resonance properties and propulsion system noise patterns, with misclassification rates consistently below 0.4%. The consistently high performances across all variants demonstrate the effectiveness of the PR-Conv mechanism at maintaining robust multi-scale feature extraction across different network capacities while validating the flexible balancing capability of the unified-width-scaling framework between performance and efficiency.

Beyond the classification performance, the unified-width-scaling framework demonstrates significant flexibility in computational resource configuration. As shown in Table 1, the MobilePR-ConvNet series presents a complete resource gradient from extreme lightweight to high performance, where the 0.25× variant requires only 0.32 MB storage and 20.664 ms latency for IoT devices, while the 0.5× to 1.0× variants scale to 0.89 MB 1.71 MB and 2.77 MB, providing differentiated solutions for mobile edge and desktop deployment. A memory gradient from 15.75 MB to 61.27 MB and power consumption from 1.54 mW to 3.99 mW validate the practical value in battery-powered scenarios.

### 4.2. Performance Comparison with Existing Methods

#### 4.2.1. Comparison with Lightweight Baseline Networks

This section aims to analyze the comparison results of the proposed method with classical lightweight networks in terms of their parameter efficiency and computational complexity. As shown in Table 2, the MobilePR-ConvNet series demonstrates significant advantages in terms of parameter efficiency and computational complexity. MobilePR-ConvNet-0.25× achieved 92.32% accuracy with only 70.39 K parameters and 23.79 M FLOPs, reducing the parameter count by 90.3% compared to SqueezeNet, with a similar parameter count, while improving the accuracy by 11.24 percentage points, while MobilePR-ConvNet-1.0× achieved 98.58% accuracy with 668.23 K parameters and 229.91 M FLOPs, reducing the parameter count by 94.0% and the computational overhead by 72.9% compared to ResNet18 while maintaining a comparable performance. In terms of the inference speed, all variants maintained GPU inference times between 2.88 ms and 5.01 ms, where MobilePR-ConvNet-1.0× with a 5.01 ms inference time is significantly lower than MobileNet V1 with 6.82 ms, despite the similar accuracy but larger parameter count. These results validate the effectiveness of the PR-Conv mechanism in lightweight network design through adaptive multi-scale feature extraction.

A comprehensive performance comparison indicates that the proposed method achieves an excellent balance between efficiency and accuracy. MobilePR-ConvNet-0.75× achieved 96.72% accuracy with only 404.31 K parameters and 139.75 M FLOPs, surpassing all other lightweight networks, including the MobileNet series and SqueezeNet, while the F1-score of 96.73% validates the model’s robustness under class-imbalanced conditions. Compared to ResNet6 with 2777.54 K parameters and 89.51% accuracy, MobilePR-ConvNet-0.75× achieved 7.21-percentage-point-higher accuracy while reducing the parameter count by 85.4%, demonstrating the ability of the PR-Conv mechanism to achieve an excellent recognition performance while maintaining extremely low computational overhead.

#### 4.2.2. Comparison with State-of-the-Art Methods

To further validate the advancement of the proposed method, this study conducted comprehensive comparisons with state-of-the-art methods recently published in the field of underwater acoustic target recognition, including the deep learning methods UATFSN [25], MSRDN [16], SCAE [2], and MSLEFC [17] and the cutting-edge attention architectures Mamba [33], Transformer [34], Conformer [35], and SKNet [26], where all methods were evaluated under identical experimental conditions to ensure the fairness and reliability of the comparison results. As shown in Table 3, the proposed method achieved the highest accuracy of 98.58%, significantly surpassing all comparison methods and improving by 0.35% over UATFSN, with the second-best performance among traditional deep learning methods, and by 15.70%, 15.64%, and 21.05% over CFTANet, MSLEFC, and SCAE, respectively, demonstrating advantages in feature extraction and model design. More notably, compared to the recently prominent cutting-edge attention architectures, the proposed method improved the accuracy by 8.76%, 10.86%, and 11.80% over Mamba, Conformer, and Transformer, respectively, fully demonstrating that path-routing convolution can more effectively capture the key discriminative features of underwater acoustic signals through the adaptive multi-scale feature extraction mechanism. Particularly noteworthy is the 5.34-percentage-point accuracy improvement over SKNet, which similarly employs multi-scale receptive-field selection, where this significant advantage stems from our use of dilated convolutions to construct multi-scale paths rather than enlarging the kernel sizes, effectively avoiding parameter redundancy while achieving broader receptive-field coverage. Even the most lightweight 0.25× variant with 92.32% accuracy still outperformed all the cutting-edge attention methods, further validating the effectiveness and superiority of the proposed architectural design.

### 4.3. Robustness Analysis

This section aims to analyze the robustness performances of the proposed models under noisy environments and data-scarce conditions.

**Figure 5:**
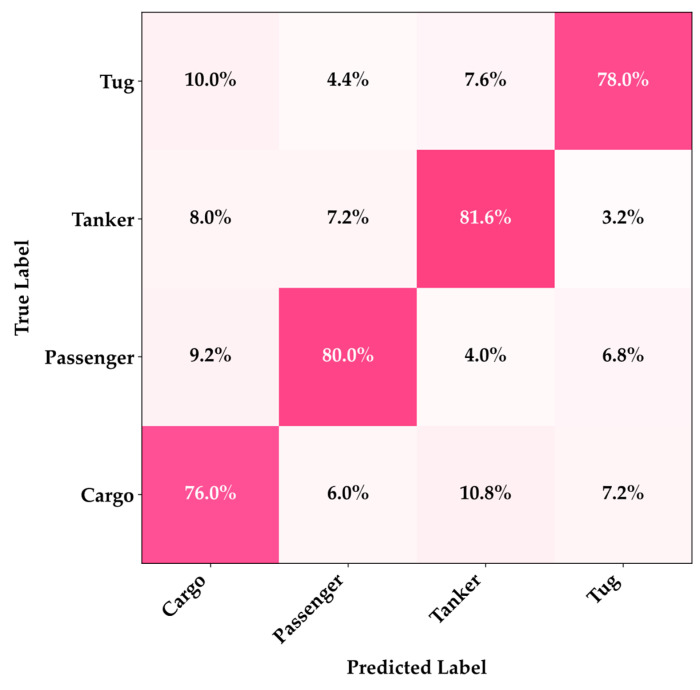
Confusion matrix of MobilePR-ConvNet-1.0× under extreme noise conditions at SNR of 10 dB, showing classification performances across four vessel categories.

As illustrated in Figure 5, the confusion matrix reveals classification details under extreme noise conditions at a signal-to-noise ratio of 10 dB, where the Cargo and Passenger categories maintained high recognition rates of 76.0% and 80.0%, demonstrating excellent noise robustness, but the 10.8% cross-confusion between Cargo and Tanker as well as the 7.2% misclassification between Tug and Passenger indicate that strong noise interference masked the low-frequency features of large-vessel engines and the mid-frequency features of small-vessel propellers, leading to blurred acoustic feature boundaries. However, even the most lightweight variant maintained an acceptable recognition performance under extremely low signal-to-noise ratio conditions, demonstrating the effectiveness of the PR-Conv adaptive multi-scale feature extraction mechanism in complex acoustic environments. These results indicate that the proposed model series possesses strong anti-interference capabilities in real marine noise environments.

**Figure 6:**
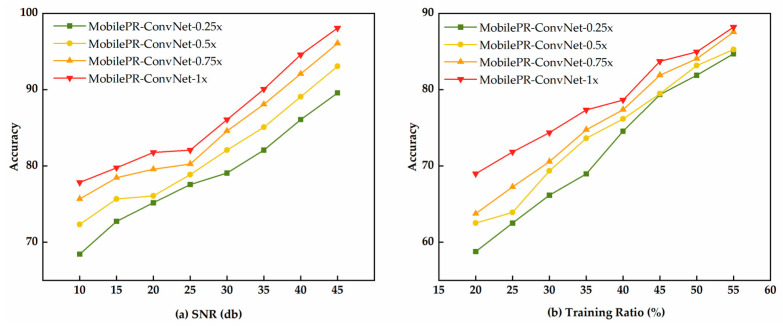
Robustness evaluation of MobilePR-ConvNet variants: (**a**) performances under different SNR conditions; (**b**) performances with varying training data ratios.

As shown in Figure 6a, all the MobilePR-ConvNet variants demonstrated excellent robustness performances in noisy environments. MobilePR-ConvNet-1.0× achieved 97.2% accuracy under high-signal-to-noise-ratio conditions of 45 dB, and as the signal-to-noise ratio decreased to the extreme noise level of 10 dB, the accuracy remained at 77.8%, while MobilePR-ConvNet-0.75× and MobilePR-ConvNet-0.25× achieved 75.2% and 68.1% accuracies, respectively, under the same conditions. As shown in Figure 6b, all models demonstrated good learning efficiencies in training-data-scarce scenarios. When the training data proportion was only 20%, MobilePR-ConvNet-1.0× achieved 69.2% accuracy, and as the data proportion increased to 60%, the accuracy improved to 87.6%, while MobilePR-ConvNet-0.75× and MobilePR-ConvNet-0.25× achieved 85.3% and 78.4% accuracies, respectively, at a 60% data proportion. All variants achieved stable performance levels when the training data proportion was 30%, where the MobilePR-ConvNet-1.0× accuracy of 74.8% validated the efficient feature learning capability of PR-Conv under data-constrained conditions. These results demonstrate that the proposed model series can maintain a reliable recognition performance in practical deployment scenarios with scarce training samples.

### 4.4. Frequency Response Analysis

**Figure 7:**
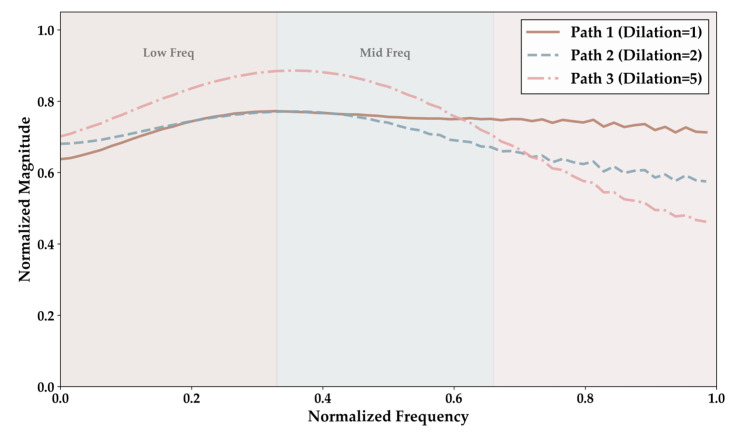
Frequency response analysis of three parallel paths in PR-Conv: normalized magnitude responses across the frequency spectrum for Path 1 (Dilation = 1), Path 2 (Dilation = 2), and Path 3 (Dilation = 5).

This section aims to demonstrate the frequency component characteristics captured by each of the three parallel paths in the PR-Conv mechanism through frequency response analysis. As shown in Figure 7, the three parallel paths in the PR-Conv mechanism exhibit differentiated frequency response characteristics. Path 1 with a dilation rate of 1 exhibits frequency response magnitudes from 0.65 to 0.78 in the low-frequency band precisely covering the primary energy distribution region of the low-frequency line spectra from the main engines of large cargo vessels, such as Cargo and Tanker. Path 2 with a dilation rate of 2 maintains stable responses from 0.70 to 0.75 in the mid-frequency band, effectively capturing the propeller blade passage frequencies and harmonic components from vessels such as Tug and Passenger. Path 3 with a dilation rate of 5 demonstrates the strongest frequency response, with a peak reaching 0.90 in the low-to-mid-frequency band integrating broadband spectral structures of Cargo and Tanker and capturing global acoustic feature patterns. Notably, all three paths exhibit attenuation trends below 0.5 in the high-frequency band, which highly aligns with the physical property that the radiated-noise energy from the four commercial vessel types is primarily concentrated in the low-to-mid-frequency range, fully validating the effectiveness of the three-path design in specifically covering the key frequency component distribution of underwater acoustic targets.

### 4.5. Hyperparameter Sensitivity Analysis

This section systematically analyzes the rationality of the key hyperparameter selection in the PR-Conv mechanism through the control variable method, where each experiment changes only a single hyperparameter while keeping the others constant. As shown in Table 4, we conducted a sensitivity analysis on the compression ratio minimum feature dimension and convolution kernel size configuration. The compression ratio controls the dimensionality of the compact feature vectors in the routing mechanism. With the minimum feature dimension fixed at 32, a kernel size of 3 × 3, and the dilation rates fixed at 1-2-5 when the compression ratio was 4, the accuracy reached 98.21% but introduced unnecessary computational overhead. When the compression ratio was 16, the accuracy decreased to 96.82%, indicating an insufficient feature representation capability, while a compression ratio of 8 achieved an optimal accuracy of 98.58%. The minimum feature dimension sets the lower bound for compact feature vectors. With the compression ratio fixed at 8, a kernel size 3 × 3, and the dilation rates fixed at 1-2-5 when the minimum feature dimension was 16, the accuracy only reached 97.34%, indicating that an excessively small dimension leads to an insufficient expressive capability. When the minimum feature dimension was 64, the accuracy dropped to 97.89%, indicating that an excessively large dimension increases the computational burden, while a minimum feature dimension of 32 maintained the highest accuracy of 98.58%, ensuring a sufficient expressive capability. Kernel size configuration determines the base receptive-field size of the three paths. With the compression ratio fixed at 8, the minimum feature dimension fixed at 32, and the dilation rates fixed at 1-2-5, the uniform small kernel 3 × 3-3 × 3-3 × 3 configuration achieved only 97.45% accuracy, indicating insufficient receptive-field coverage. The large kernel 5 × 5-5 × 5-5 × 5 configuration achieved 97.12% accuracy but significantly increased the parameters, leading to reduced computational efficiency, while our 3 × 3 kernel with the dilated convolution scheme achieved an optimal accuracy of 98.58%, maintaining parameter efficiency while flexibly expanding equivalent receptive fields through dilation rates of 1-2-5.

### 4.6. Ablation Study

To systematically validate the effectiveness of each core operation in PR-Conv, we conducted ablation experiments to evaluate the impact of the splitting, fusion, and routing mechanisms on the recognition performance. The experimental settings included the single-path baseline, two-path configuration, and three-path schemes with different dilation rates; the fixed-weight scheme without routing; and standard convolution alternatives. Table 5 presents the performance comparison of the configurations. All experiments were conducted under identical training conditions to ensure a fair comparison.

The single-path baseline achieved only 83.15% accuracy, the two-path configuration significantly improved to 96.97%, and the complete three-path scheme further reached the optimal performance of 98.58%, validating the necessity of the multi-path splitting operation for covering multi-scale spectral features, where the 1.61-percentage-point improvement from the two-path to three-path scheme indicates that adding the medium-scale path effectively fills the coverage gap between local and global features. The dilation rate comparison shows that the dense configuration achieved 97.23%, while the sparse configuration reached 96.54%, both lower than our balanced configuration of 98.58%, demonstrating that the fusion operation requires balancing the coverage range and feature redundancy. Removing the routing mechanism reduced the performance to 91.73%, a drop of 6.85 percentage points compared to the complete scheme, indicating the critical role of the routing operation in dynamically selecting the optimal scale combinations. The standard convolution alternative reduced the performance to 92.85%, further validating the importance of dilated convolutions in enlarging receptive fields.

### 4.7. Cross-Dataset Validation on ShipsEar

To validate the generalization capability of the proposed method, we conducted cross-dataset validation experiments on the publicly available ShipsEar dataset. The dataset was segmented according to 5 s durations, obtaining 2223 independent acoustic sample segments; the original 12 target categories were regrouped into four main categories, with Class A covering fishing boat, trawler, mussel boat, tugboat, and dredger; Class B including motorboat, pilot boat, and sailboat; Class C including passengers; and Class D including ocean liner and RO-RO. The dataset adopted the same 8:2 training–test set division ratio as that of DeepShip, ensuring consistency in the experimental setup.

As shown in Table 6, the proposed method demonstrated an excellent cross-dataset generalization performance on the ShipsEar dataset. The accuracies of the four network variants increase progressively from 92.47% to 97.82%, indicating that the serialized architecture design can effectively adapt to different capacity requirements. Notably, despite the relatively limited sample size and significant differences in the category composition of the ShipsEar dataset, the proposed method still achieved high accuracy of 97.82%, fully validating the robustness and generalization capability of the PR-Conv mechanism in different underwater acoustic target recognition scenarios.

## 5. Conclusions

This paper proposes a novel lightweight underwater acoustic target recognition method based on path-routing convolution (PR-Conv), achieving the accurate identification of surface-navigating vessels by analyzing the time–frequency characteristics of underwater acoustic signals, effectively solving the technical bottleneck of deploying deep learning models on resource-constrained underwater devices. The MobilePR-ConvNet-series models built upon the PR-Conv mechanism achieved significant engineering application results on the DeepShip dataset, where the model series achieved a 98.58% recognition accuracy with at most 668.23 K parameters, reducing the parameter count by 94.0% compared to ResNet18 while maintaining a comparable performance and surpassing all lightweight baseline networks, including the MobileNet series and SqueezeNet. In comparison with the latest state-of-the-art (SOTA) methods, the proposed models demonstrated clear advantages, improving the accuracy by 0.35% over UATFSN among the SOTA methods and by 8.76%, 10.86%, and 11.80% over Mamba, Conformer, and Transformer, respectively. Under practical deployment conditions, the proposed models exhibited excellent environmental adaptability, achieving a maximum accuracy of 77.8% under extremely low signal-to-noise ratio conditions of 10 dB and approaching 70% accuracy with only 15% training data, fully validating the robustness of the method in complex marine environments. Cross-dataset generalization experiments further demonstrated the effectiveness of the proposed method, achieving 97.82% accuracy on the ShipsEar dataset. Frequency response analysis indicates that PR-Conv effectively captures differentiated frequency components of vessel-radiated noise in the low-to-mid-frequency range through three parallel paths with dilation rates of 1, 2, and 5, providing a new technical approach for underwater target feature extraction. Future research can explore embedding PR-Conv as a local feature extractor into Transformer or hybrid attention architectures, providing new solutions for more complex underwater sensing tasks, such as underwater acoustic target localization and tracking.

## Figures and Tables

**Table 1:** Model size and computational resource usage comparison.

Model	Size (MB)	Latency (ms)	FPS (single)	CPU Mem (MB)	Power (mW)	CPU Util (%)
MobilePR-ConvNet-0.25×	0.32	20.664	48.39	15.75	1.54	442.9
MobilePR-ConvNet-0.5×	0.89	32.091	31.16	31.65	2.47	650.5
MobilePR-ConvNet-0.75×	1.71	75.854	13.18	45.95	3.00	543.1
MobilePR-ConvNet-1.0×	2.77	85.787	11.66	61.27	3.99	675.1

**Table 2:** Comprehensive performance comparison between proposed MobilePR-ConvNet variants and baseline methods.

Model	Params	FLOPs	GPU Inference (ms)	Macro-Accuracy (%)	Macro-F1 Score (%)
MobilePR-ConvNet-0.25×	70.39 K	23.79 M	2.88	92.32	92.35
MobilePR-ConvNet-0.5×	205.03 K	71.37 M	3.84	94.56	94.71
MobilePR-ConvNet-0.75×	404.31 K	139.75 M	4.67	96.72	96.73
MobilePR-ConvNet-1.0×	668.23 K	229.91 M	5.01	98.58	98.58
ResNet6	2777.54 K	647.87 M	3.82	89.51	89.12
ResNet18	11,172.29 K	849.44 M	4.62	98.58	98.56
MobileNet V1	3210.51 K	283.13 M	6.82	86.32	86.23
MobileNet V2	2228.42 K	318.99 M	5.82	90.31	89.77
MobileNet V3	1515.31 K	5.128 M	4.92	91.45	91.13
SqueezeNet	728.07 K	316.30 M	2.31	81.08	81.31

**Table 3:** Performance comparison with state-of-the-art methods on DeepShip dataset.

Method	Accuracy (%)
UATFSN	98.23
MSRDN	85.20
SCAE	77.53
MSLEFC	82.94
Mamba	89.82
Transformer	86.78
Conformer	87.72
SKNet	93.24
MobilePR-ConvNet-0.25×	92.32
MobilePR-ConvNet-0.5×	94.56
MobilePR-ConvNet-0.75×	96.72
MobilePR-ConvNet-1.0×	98.58

**Table 4:** Sensitivity analysis of key hyperparameters using control variable method.

Parameter	Value	Fixed Conditions	Accuracy (%)
Compression Ratio	4	D_min = 32; Kernel = 3 × 3; Dilation = 1-2-5	98.21
	8 (ours)	D_min = 32; Kernel = 3 × 3; Dilation = 1-2-5	98.58
	16	D_min = 32; Kernel = 3 × 3; Dilation = 1-2-5	96.82
Minimum Dimension	16	r = 8; Kernel = 3 × 3; Dilation = 1-2-5	97.34
	32 (ours)	r = 8; Kernel = 3 × 3; Dilation = 1-2-5	98.58
	64	r = 8; Kernel = 3 × 3; Dilation = 1-2-5	97.89
Kernel Size Config.	[3 × 3-3 × 3-3 × 3]	r = 8; D_min = 32; Dilation = 1-2-5	97.45
	[3 × 3 + Dilation] (ours)	r = 8; D_min = 32; Dilation = 1-2-5	98.58
	[5 × 5-5 × 5-5 × 5]	r = 8; D_min = 32; Dilation = 1-2-5	97.12

**Table 5:** Results of ablation study on core operations of PR-Conv.

Configuration	Operation Validated	Dilation Rates	Accuracy (%)
Single path	Splitting	1	83.15
Two paths	Splitting	1, 5	96.97
Three-path dense	Fusion	1, 2, 3	97.23
Three-path sparse	Fusion	1, 3, 7	96.54
Three-path w/o routing	Routing	1, 2, 5	91.73
Three-path w/o dilation	Receptive field	Standard conv	92.85
Three-path complete	All operations	1, 2, 5	98.58

**Table 6:** Results of cross-dataset validation on ShipsEar dataset.

Network Variant	Width Multiplier	Accuracy (%)
MobilePR-ConvNet-0.25×	0.25×	92.47
MobilePR-ConvNet-0.5×	0.50×	94.63
MobilePR-ConvNet-0.75×	0.75×	96.28
MobilePR-ConvNet-1.00×	1.00×	97.82

## Data Availability

The data that has been used is confidential.

## Abbreviations

The following abbreviations are used in this manuscript:

PR-Conv	Path-routing convolution
MFCCs	Mel-frequency cepstral coefficients
STFT	Short-time Fourier transform
